# Rates of Acute Kidney Injury Utilizing Area Under the Concentration–Time Curve Versus Trough-Based Vancomycin Dosing Strategies in Patients With Obesity

**DOI:** 10.1093/ofid/ofaf205

**Published:** 2025-04-03

**Authors:** Corey M Guidry, Emily A Siegrist, Stephen B Neely, Lyndee Springer, Bryan P White

**Affiliations:** Department of Pharmacy: Clinical and Administrative Sciences, University of Oklahoma College of Pharmacy, Oklahoma City, Oklahoma, USA; Department of Pharmacy, OU Health, Oklahoma City, Oklahoma, USA; Department of Pharmacy: Clinical and Administrative Sciences, University of Oklahoma College of Pharmacy, Oklahoma City, Oklahoma, USA; Department of Pharmacy, United States Public Health Service Lawton Indian Hospital, Lawton, Oklahoma, USA; Department of Pharmacy, OU Health, Oklahoma City, Oklahoma, USA

**Keywords:** acute kidney injury, area under the curve, nephrotoxicity, obese, vancomycin

## Abstract

**Background:**

Vancomycin is commonly utilized for the treatment of methicillin-resistant *Staphylococcus aureus* (MRSA) infections. Dosing recommendations for vancomycin have shifted in recent years to favor area under the concentration–time curve (AUC) instead of trough-based dosing strategies to decrease vancomycin exposure and rates of acute kidney injury (AKI). However, little data exist on the safety and efficacy of AUC-based dosing in patients with obesity.

**Methods:**

This was a single-center retrospective cohort study conducted between 1 January 2014 and 31 December 2022. Adult patients aged ≥18 years were included if they were obese and received vancomycin for treatment of a severe MRSA infection for at least 72 hours. The primary outcome was incidence of AKI based on Kidney Disease: Improving Global Outcomes (KDIGO) criteria.

**Results:**

After initial screening, 398 patients were included, with 230 in the trough group and 168 in the AUC group. Rates of AKI were lower in the AUC group compared to the trough group (11.3% vs 25.2%, *P* < .001). After adjusting for potential confounders, logistic regression maintained a reduction in AKI with AUC-based dosing for cumulative doses less than the median of 10 250 mg (odds ratio, 0.47 [95% confidence interval, .25–.88]) but not for doses above. Rates of initial target attainment were also higher with AUC-based dosing (50.0% vs 23.9%, *P* < .001).

**Conclusions:**

Patients with obesity receiving vancomycin for treatment of severe MRSA infections experienced lower rates of AKI when utilizing an AUC- versus trough-based dosing strategy.

## Teaser

Patients with obesity receiving vancomycin for treatment of severe methicillin-resistant *Staphylococcus aureus* infections experienced lower rates of acute kidney injury, higher initial target attainment, and decreased overall vancomycin exposure when utilizing area under the concentration–time curve versus a trough-based dosing strategy.

Vancomycin remains a first-line option for the treatment of severe methicillin-resistant *Staphylococcus aureus* (MRSA) infections [1]. However, vancomycin therapeutic monitoring recommendations have shifted in recent years. Vancomycin therapeutic monitoring consensus guidelines changed from previously recommending trough-based monitoring to area under the concentration–time curve (AUC)–guided monitoring [2]. This change was based on a body of literature suggesting that AUC/minimum inhibitory concentration of 400–600 mg/L-hour is associated with improved clinical response and reduced nephrotoxicity compared to trough-based dosing targeting troughs of 15–20 mg/L [2–4].

For obese patients, AUC-guided monitoring is recommended, but optimal empiric dosing to target AUCs in this population is poorly defined, as there are limited data evaluating the impact of AUC-based dosing strategies on rates of acute kidney injury (AKI) and AUC target attainment in obese patients [2, 5]. This is of particular clinical significance as the prevalence of obesity in the United States rises, with estimates that more than half of the US population will be overweight or obese in the next 10 years [6].

Vancomycin pharmacokinetics in obesity is dissimilar to that in nonobese patients in several ways. Clearance of vancomycin tends to increase with body weight, while volume of distribution per kilogram total body weight tends to be lower in obese patients [2, 7, 8]. Obese patients tend to have higher rates of vancomycin-related AKI and more levels outside of target range, especially when using dosing based on total body weight [9–13]. Several different strategies for initial dosing regimens have been proposed [2]. Crass et al proposed an initial AUC-based dosing strategy for obese patients, which uses allometric scaled total body weight, age, serum creatinine (SCr), and sex to better estimate vancomycin clearance [14].

Previous studies have evaluated the impact of transitioning from trough- to AUC-based monitoring in obese subjects with a body mass index (BMI) >30 kg/m^2^. One study demonstrated decreased rates of AKI, particularly in patients with BMI >40 kg/m^2^, and found that lower total daily doses of vancomycin were used, consistent with previous literature [5]. However, the achievement of therapeutic range was not discussed. A second study utilized first-dose kinetics on all AUC patients, and did showed increased target attainment [15].

Following the update in vancomycin monitoring guidelines, our institution switched from trough-based dosing to AUC-guided dosing. This study aims to evaluate the rate of AKI in obese patients after switching from a trough-based to AUC-guided dosing. Secondary aims were to compare vancomycin total daily doses and achievement of therapeutic targets with trough and AUC dosing strategies.

## METHODS

### Study Design and Patient Selection

We completed a retrospective study examining outcomes following a change in dosing protocol at a 500-bed academic medical center. This study was designated as exempt by the institutional review board; a waiver of informed consent was granted due to the retrospective design. A report was developed from the electronic medical record (EMR) containing all patients admitted to our institution between 1 January 2014 and 31 December 2022 with 1 or more *International Classification of Diseases*, *Ninth Revision* or *Tenth Revision* codes for obesity who received vancomycin via a pharmacy-driven dosing protocol. In August 2019, our institution switched from a trough-based vancomycin dosing protocol to an AUC-based protocol. All pharmacists received live training on this change. The policy was also edited in August 2022 to allow pharmacists to order MRSA nasal swabs. Therefore, all patients admitted to prior to 1 August 2019 were placed in the pre-protocol change “trough” cohort, and (to allow for washout) all those admitted on or after 1 September 2019 were placed in the post-protocol change “AUC” cohort. For calculation of empiric dosing regimen in obese patients, our institutional protocol uses the equations suggested by Crass et al. For dose adjustments, 2 levels are drawn and AUC is calculated using trapezoidal pharmacokinetics [16].

Criteria for inclusion were age ≥18 years, a BMI of at least 30 kg/m^2^, and receipt of vancomycin per a “pharmacy to dose” protocol for a severe MRSA infection (eg, sepsis, bacteremia, pneumonia, osteomyelitis). Patients who received vancomycin for at least 72 hours with a trough goal of 15–20 mg/L (“trough” cohort) or targeted AUC value of 400–600 mg/L-hour (“AUC” cohort) were placed in those respective cohorts. Patients also had to have at least 1 or more vancomycin levels drawn appropriately (eg, trough levels drawn within 1 hour prior to infusion of the next vancomycin dose and peak levels drawn at least 2 hours after completion of vancomycin infusion) to interpret or calculate target attainment. Patients were excluded if they had documented receipt of vancomycin at an outside facility, were pregnant or breastfeeding, or had a diagnosis of cystic fibrosis. Patients treated with vancomycin for cellulitis or urinary tract infection were excluded, as these nonsevere infections are treated with lower vancomycin trough goals (ie, 10–15 mg/L) at our institution. Patients treated for meningitis or other suspected infection of the central nervous system were also excluded, as these patients do not qualify for AUC-based dosing per the new institutional protocol. Additionally, patients were excluded if they developed AKI prior to vancomycin initiation during the same hospitalization, had a baseline creatinine clearance (CrCl) of ≤30 mL/minute, or were on any form of renal replacement therapy, as these patients also do not qualify for AUC-based dosing per our institutional protocol.

### Data Collection and Outcomes

The EMR was utilized to collect demographic data including age, sex, race, weight, height, comorbid conditions, and SCr values throughout admission. Information regarding vancomycin regimens was also collected, including dosing and administration times, peak and/or trough vancomycin concentrations, and total duration of therapy. Documentation within the EMR was utilized to assess rates of initial target attainment. Target attainment was defined as a vancomycin trough level between 15 and 20 mg/L (“trough” cohort) or calculated AUC value between 400 and 600 mg/L-hour (“AUC” cohort), using the first value(s) available at steady-state (ie, after receipt of at least 3 doses of vancomycin). In cases of missing documentation, peak and/or trough values (drawn appropriately at steady-state) were utilized to assess (ie, trough goal) or calculate (ie, AUC goal) initial target attainment. Receipt of concomitant nephrotoxic agents (if administered within 24 hours of vancomycin) was also collected. Applicable agents included loop diuretics, angiotensin-converting enzyme inhibitors/angiotensin receptor blockers, nonsteroidal anti-inflammatory drugs, iodinated contrast, selected antimicrobials (eg, aminoglycosides, amphotericin B, piperacillin-tazobactam, trimethoprim-sulfamethoxazole), calcineurin inhibitors, and selected chemotherapeutic agents (eg, cisplatin, ifosfamide).

The primary outcome of this study was the incidence of AKI in the AUC cohort as compared to the trough cohort. We defined AKI based on the Kidney Disease: Improving Global Outcomes (KDIGO) criteria of an increase in SCr of ≥0.3 mg/dL within 48 hours and/or an increase in SCr to ≥1.5 times baseline within 7 days [17]. Secondary outcomes included all-cause inpatient mortality and incidence of nephrotoxicity based on the Acute Dialysis Quality Initiative (ADQI) Group “injury” stage (an increase in SCr to ≥2 times baseline) [18]. To identify occurrence of AKI, the initial SCr value just prior to vancomycin initiation was collected and served as a baseline. If no value was available prior to initiation of vancomycin, then the first available value after initiation was utilized. Then, the greatest SCr values within 48 hours, 7 days, and any time after vancomycin initiation were collected. If vancomycin was discontinued after at least 72 hours of therapy, only SCr values up to 7 days after discontinuation were utilized to assess AKI. Based on requisite increases in SCr, patients could meet criteria for AKI for both the primary and secondary outcomes. Patients who did not meet 1 or more of the previous definitions of AKI were classified as having no AKI.

### Statistical Analysis

Descriptive statistics were used to summarize patient demographics and clinical characteristics. Categorical data were analyzed using χ^2^ or exact tests and continuous variables using Mann-Whitney *U* tests. A multivariable logistic regression analysis was performed to assess the adjusted odds (95% confidence interval) of AKI occurring. Backwards selection was used for candidate variables of interest in the model with them entering if *P* < .2 and retained if *P* < .05. Model fit and diagnostics were examined. The categorical by continuous interaction term of dose cohort and cumulative vancomycin dose was included and reported as the adjusted odds of AKI between dosing cohorts at the 10th, 25th, 50th, 75th, and 90th percentiles of cumulative vancomycin dose. Data were collected in REDCap and the analyses were completed using SAS version 9.4 software (SAS Institute Inc, Cary, North Carolina), with an a priori α set to .05.

## RESULTS

**Figure 1.**
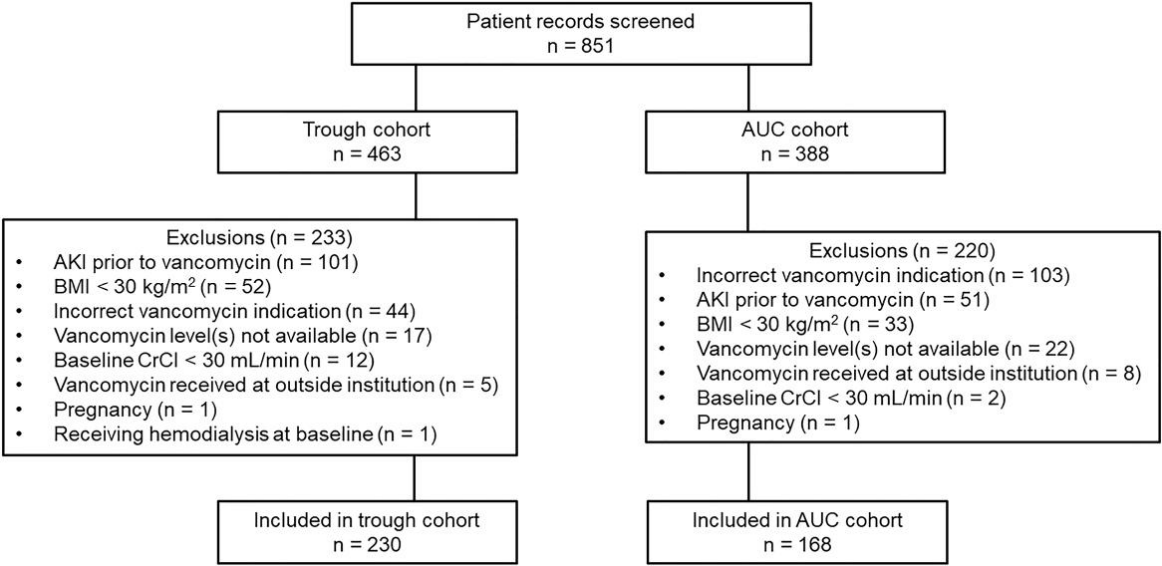
Flowchart for included patients. Abbreviations: AKI, acute kidney injury; AUC, area under the concentration–time curve; BMI, body mass index; CrCl, creatinine clearance.

A total of 851 patients were screened and 398 were included in the final analysis (Figure 1). Though a higher percentage of male patients was included in the AUC cohort compared to the trough cohort (51.8% vs 41.7%, respectively), all other baseline characteristics were similar between the 2 groups (Table 1). Patients were primarily White (74%) and received care on a general medicine floor (78%). Overall, included patients had a median body weight of 118 kg and BMI of 40 kg/m^2^. Receipt of nephrotoxins was fairly consistent, though iodinated contrast was given significantly more to patients in the AUC cohort (12.5% vs 3.0%, *P* < .001).

**Table 1.** Baseline Characteristics

Characteristic	Trough (n = 230)	AUC (n = 168)	*P* Value
Sex, male	96 (41.7)	87 (51.8)	.047
Age, y, median (IQR)	52 (41–61)	54 (46–63)	.115
Race			.140
White	167 (72.6)	127 (75.6)	
Black or African American	36 (15.7)	17 (10.1)	
American Indian/Alaska Native	15 (6.5)	7 (4.2)	
Asian	3 (1.3)	3 (1.8)	
Native Hawaiian/other Pacific Islander	1 (0.4)	1 (0.6)	
Unknown/not reported/>1	8 (3.5)	13 (7.7)	
Total body weight, kg, median (IQR)	120 (102–139)	116 (106–135)	.599
BMI, kg/m^2^, median (IQR)	40.8 (35.9–45.8)	39.4 (35.0–46.1)	.308
Baseline SCr, mg/dL, median (IQR)	0.84 (0.69–0.98)	0.89 (0.69–1.07)	.162
Care location			.061
General medicine floor	187 (81.3)	122 (72.6)	
Intensive care unit	26 (11.3)	33 (19.6)	
Emergency department	17 (7.4)	13 (7.7)	
Length of stay, d, median (IQR)	11 (7–19)	11 (7–19.5)	.368
CCI score, median (IQR)	5 (2–8)	5.5 (3–8.5)	.167
History of myocardial infarction	24 (10.4)	26 (15.5)	.134
History of congestive heart failure	73 (31.7)	64 (38.1)	.187
History of diabetes	133 (57.8)	101 (60.1)	.424
History of renal disease	46 (20)	31 (18.5)	.699
History of cancer	75 (32.6)	47 (28)	.418
Concomitant nephrotoxin receipt			
Loop diuretic	104 (45.2)	63 (37.5)	.123
ACEi/ARB	73 (31.7)	49 (29.2)	.583
NSAID	51 (22.2)	36 (21.4)	.859
Iodinated contrast	7 (3.0)	21 (12.5)	<.001
Selected antimicrobial	144 (62.6)	89 (53.0)	.054
Calcineurin inhibitor	1 (0.4)	1 (0.6)	1.000
Selected chemotherapy	0 (0)	0 (0)	

Data are reported as No. (%) unless otherwise indicated.

Abbreviations: ACEi, angiotensin-converting enzyme inhibitor; ARB, angiotensin receptor blocker; AUC, area under the concentration–time curve; BMI, body mass index; CCI, Charlson Comorbidity Index; IQR, interquartile range; NSAID, nonsteroidal anti-inflammatory drug; SCr, serum creatinine.

The primary outcome of AKI per KDIGO criteria occurred in 58 patients (25.2%) in the trough cohort and 19 patients (11.3%) in the AUC cohort (*P* < .001). Rates of AKI based on the ADQI criteria for “injury” were also significantly higher in the trough cohort, though no differences in all-cause mortality were identified (Table 2).

**Table 2.** Primary and Secondary Outcomes

Outcome	Trough (n = 230)	AUC (n = 168)	*P* Value
Primary outcome, No. (%)			
AKI (KDIGO definition)	58 (25.2)	19 (11.3)	<.001
Secondary outcomes, No. (%)			
AKI (ADQI definition)	27 (11.7)	9 (5.4)	.028
All-cause mortality	15 (6.5)	12 (7.1)	.808

Abbreviations: ADQI, Acute Dialysis Quality Initiative; AKI, acute kidney injury; AUC, area under the concentration–time curve; KDIGO, Kidney Disease: Improving Global Outcomes.

Data regarding vancomycin regimens received by patients are reported in Table 3. Compared to those in the trough cohort, patients in the AUC cohort were more likely to receive a loading dose (57.8% vs 81.5%, *P <* .0001), though no differences were seen in the total or weight-based (calculated using actual body weight) amount of vancomycin received. Patients in the AUC cohort had significantly higher rates of initial target attainment (50% vs 23.9%) as well as lower initial vancomycin trough values (12.9 vs 14.8 mg/L). Vancomycin dosing was significantly lower in the AUC cohort compared to the trough cohort, with patients receiving a median of 1625 mg less vancomycin in the initial 72 hours of therapy. The median duration of vancomycin therapy was 121 hours, with no significant difference between cohorts.

**Table 3.** Vancomycin Regimen Data

Characteristic	Trough (n = 230)	AUC (n = 168)	*P* Value
Loading dose administered, No. (%)	133 (57.8)	137 (81.5)	<.0001
Total loading dose, mg	2500 (2000–3000)	2500 (2500–2750)	.511
Weight-based loading dose, mg/kg	22.8 (19.8–24.7)	22.7 (19–24)	.293
Initial target attainment, No. (%)			
Yes	55 (23.9)	84 (50)	<.0001
No, subtherapeutic	59 (25.7)	48 (28.6)	
No, supratherapeutic	117 (50.9)	35 (20.8)	
Initial vancomycin trough, mg/L	14.8 (10.8–20.2)	12.9 (8.9–17.8)	.036
Cumulative vancomycin dose, mg			
Overall (0–72 h)	11 125 (8750–13 000)	9500 (7875–11 000)	<.0001
0–24 h	4500 (3500–5500)	4250 (3750–5000)	.168
24–48 h	3500 (2000–4000)	2500 (1750–3500)	<.0001
48–72 h	3500 (2000–4250)	2500 (1750–3375)	<.0001
Duration of vancomycin therapy, h	117.4 (87–185.3)	131.5 (98.5–186)	.116

Data are reported as median (interquartile range) unless otherwise indicated.

Abbreviation: AUC, area under the concentration–time curve.

Logistic regression was employed to analyze the relationship between the incidence of AKI and vancomycin dosing strategy (Table 4). Several candidate variables were not significant in the final model and thus not included (length of hospital stay, admission CrCl, receipt of contrast dye, age, and duration of vancomycin therapy). Considering all variables in the final model, the adjusted odds of AKI were 8.9% higher for any 1 μg/mL increase in vancomycin trough, 216.8% higher for those with moderate to severe chronic kidney disease (CKD) versus none, and 34.2% lower for any 0.1 mg/dL increase in baseline SCr. Additionally, both the simple effects of dosing cohort and cumulative vancomycin dose administered within the first 72 hours of therapy and their interaction were significantly associated with AKI. Patients in the AUC dosing cohort had 82.7%, 72.5%, and 53.4% lower adjusted odds of AKI compared to the trough-based dosing cohort when receiving doses of 6500, 8250, and 10 250 mg within the first 72 hours, respectively. No difference in odds were noted between dosing groups at 12 250 and 14 500 mg.

**Table 4.** Multivariable Logistic Regression Analysis

Variable	Comparison	Reference Group	aOR (95% CI) of AKI (KDIGO Definition)^a^	*P* Value
First vancomycin trough level	Any + 1 μg/mL	Mean = 15.5	1.09 (1.05–1.13)	<.0001
Moderate to severe CKD^b^	Yes = 19.3%	No = 80.7%	3.17 (1.55–6.46)	.002
Serum creatinine prior to vancomycin initiation	Any +0.1 mg/dL	Mean = 0.83	0.66 (.57–.75)	<.0001
Dosing cohort	AUC = 42.2%	Trough = 57.8%	0.03 (.01–.37)	.006
Cumulative vancomycin dose (mg) administered in the first 72 h	Any +250 mg	Mean = 2980.5	0.96 (.93–.99)	.007
Interaction of dosing cohort and cumulative vancomycin dose			1.0003 (1.0001–1.0005)	.032
Comparison of dosing cohorts at various levels of cumulative vancomycin dose (mg) administered in the first 72 h				
6500 mg (10th percentile)	AUC = 42.2%	Trough = 57.8%	0.17 (.06–.48)	.0003
8250 mg (25th percentile)	AUC = 42.2%	Trough = 57.8%	0.28 (.13–.57)	.0003
10 250 mg (50th percentile)	AUC = 42.2%	Trough = 57.8%	0.47 (.25–.88)	.0126
12 250 mg (75th percentile)	AUC = 42.2%	Trough = 57.8%	0.79 (.34–1.85)	.5783
14 500 mg (90th percentile)	AUC = 42.2%	Trough = 57.8%	1.43 (.40–5.18)	.6013

Abbreviations: AKI, acute kidney injury; aOR, adjusted odds ratio; AUC, area under the concentration–time curve; CI, confidence interval; CKD, chronic kidney disease; KDIGO, Kidney Disease: Improving Global Outcomes.

^a^In addition to the variables listed above, length of stay, admission creatinine clearance, receipt of contrast dye, age, and duration of vancomycin therapy were considered for the multivariable model using backward selection but not included in the final model (entered if *P* < .20 and retained if *P* < .05).

^b^As defined in the Charlson Comorbidity Index (ie, creatinine >3 mg/dL, in dialysis, status post–kidney transplant, and/or uremia).

## DISCUSSION

In this study we investigated the rate of AKI, vancomycin doses given, and initial target attainment in obese patients when switching from a trough-based to AUC-guided dosing. Our results show that AUC dosing was associated with a 55% decrease in rates of AKI. Additionally, compared to the trough-based monitoring group, the median vancomycin dose among patients in the AUC group was 15% lower in the first 72 hours of therapy.

Other studies have shown decreases in AKI in obese patients using AUC dosing for vancomycin as compared to trough-based dosing. D’Amico et al (N = 1024) reported decreases in AKI (22.7% vs 16.3%) when comparing trough versus AUC dosing, respectively [5]. The median initial trough value in the AUC group of 15.02 mg/L was higher than in our patients, and initial target attainment was not evaluated. Additionally, our patient population was more diverse (74% White vs 94%), sicker at baseline (median Charlson Comorbidity Index score, 5 vs 3), received loading doses more frequently (68% vs 22%), and had larger body habitus (120 kg vs 105 kg; BMI of 40 vs 35 kg/m^2^) than in the D’Amico et al study. Similar to other studies [19, 20], we found that history of CKD and increased vancomycin trough were risk factors for AKI. Wolfe et al (N = 254) showed decreased AKI (6.3% vs 17.4%, *P* = .035) and increased target attainment (73.6% vs 25.4%, *P* < .001) when AUC dosing was used as compared to trough dosing in obese patients. They used first-dose kinetics for the AUC calculation, which may not be feasible in all settings, and included all patients on vancomycin, not just those with trough goals of 15–20 mg/L [15]. Of note, we found increased vancomycin doses to be protective for AKI, though multiple other studies [21–23] have shown vancomycin dose as a direct risk factor for AKI. The vast majority of patients in the trough group did not achieve initial targets and nearly 51% of trough levels were supratherapeutic. Doses were likely decreased or held at 48–72 hours to correct for supratherapeutic levels, which may partially account for this finding. Additionally, it is possible that patients who were at high risk of (or had already developed) AKI were dosed more conservatively during this timeframe.

The updated vancomycin guidelines recommended a switch to AUC from trough-based dosing to decrease vancomycin exposure as well as the risk of nephrotoxicity [2]. We have shown that, in obese patients, using the vancomycin clearance equation by Crass et al meets that objective. Multiple other models exist to estimate vancomycin clearance in adults [7, 24, 25]. Although other models have demonstrated slightly improved outcomes in obese patients when compared to Crass et al [26], they require the use of more complex programs that are not compatible with homemade software used for AUC calculations by many institutions [11]. With the positive findings of our data and the ease of implementing the Crass et al equations, other institutions should investigate using the Crass et al equation for vancomycin dosing in their obese patients.

As drug costs have decreased over time, there has been debate in the literature about moving away from vancomycin to linezolid and daptomycin to ease the burden of monitoring and nephrotoxicity [27, 28]. Use of daptomycin instead of vancomycin in appropriate infections has the potential to decrease overall inpatient costs as well as provide time savings related to oversight of dosing and monitoring [29]. The largest concern with using these new agents is barriers to resistance. Daptomycin resistance in vancomycin-resistant enterococci [30] and linezolid resistance in *Staphylococcus* species [31, 32] has been reported more commonly than vancomycin resistance in *S aureus* [33], meaning that vancomycin still has a role to play in the management of MRSA infections. Utilizing optimized dosing strategies in obese patients will help improve its safety.

Our study was not without limitations. It was performed at a single center and was retrospective and observational. Any change in institutional protocol may have unintended effects on clinical practice (eg, obtaining laboratory values and drug concentrations more frequently, dosing vancomycin less “aggressively”), especially when the intent of protocol change is to decrease nephrotoxicity. An interrupted time series should be used in future studies to examine AKI trends over smaller time periods versus our pre- versus postanalysis. Somewhat counterintuitively, we found that decreasing SCr was an independent risk factor for AKI. As AKI definitions are dependent on a percent increase in SCr from baseline, this is likely because a smaller absolute increase is needed for patients with lower baseline SCr to meet the definition of AKI [17]. Therefore, rates of AKI might have been overrepresented in patients with lower baseline SCr values. Additionally, our study did not investigate any association with dosing strategy and temporality of AKI. Though the median duration of vancomycin therapy was similar between groups, it is possible that dosing strategy might have influenced the timepoint in therapy in which patients developed AKI. Similarly, as there is no universal definition of which drugs are considered “nephrotoxins,” this author group developed a list of drugs/classes to include as concomitant nephrotoxins based on previous data and expert opinion. As an example, receipt of vasopressors was not collected; though there is historical association of receipt of vasopressors and development of AKI, contemporary data suggest that this may be of less concern [34, 35]. Last, our study period of 2014–2022 covers before and during the coronavirus disease 2019 pandemic, where multiple other changes in healthcare could confound our results.

In conclusion, patients with obesity on vancomycin were less likely to have AKI, had higher target attainment, and received less vancomycin when using AUC-guided dosing based on the Crass et al equation as compared to trough-based dosing.

## Notes

***Author contributions.*** C. M. G. and B. P. W.: Conceptualization, methodology, investigation, writing–original draft/review and editing. E. A. S.: Conceptualization, methodology, writing–original draft/review and editing. S. B. N.: Formal analysis, writing–original draft/review and editing. L. S.: Investigation, writing–review and editing.

***Acknowledgments.*** The authors acknowledge Ashley Thumann and Arnold Kanagwa at the University of Oklahoma Health Sciences Center Clinical Research Data Warehouse for their assistance in identifying the original patient list for this study.

***Ethics approval.*** This study received ethical approval from the University of Oklahoma Health Sciences System Institutional Review Board (IRB) (approval number 15791) on 11 April 2023. This is an IRB-approved retrospective study; all patient information was de-identified and patient consent was not required. Patient data will not be shared with third parties.

***Potential conflicts of interest.*** B. P. W. has received research support from Shionogi and is on the speaker's bureau for Melinta. All other authors report no potential conflicts of interest.
